# Recent progress in the study of the interactions of amphotericin B with cholesterol and ergosterol in lipid environments

**DOI:** 10.1007/s00249-014-0983-8

**Published:** 2014-08-31

**Authors:** Daniel Michał Kamiński

**Affiliations:** Department of Chemistry, University of Life Sciences in Lublin, Akademicka 15, 20-950 Lublin, Poland

**Keywords:** Amphotericin B, Cholesterol, Ergosterol, Lipids, Aggregation, Pore formation, Review

## Abstract

In the past decade substantial progress has been made in understanding the organization and biological activity of amphotericin B (AmB) in the presence of sterols in lipid environments. This review concentrates mainly on interactions of AmB with lipids and sterols, AmB channel formation in membranes, AmB aggregation, AmB modifications important for understanding its biological activity, and AmB models explaining its mechanism of action. Most of the reviewed studies concern monolayers at the water–gas interface, monolayers deposited on a solid substrate by use of the Langmuir–Blodgett technique, micelles, vesicles, and multi-bilayers. Liposomal AmB formulations and drug delivery are intentionally omitted, because several reviews dedicated to this subject are already available.

## Introduction

Amphotericin B (AmB) is a macrolide polyene antifungal antibiotic (Gallis et al. 1990; AbuSalah 1996; Hartsel and Bolard 1996; Carrillo-Munoz et al. 2006; Cereghetti and Carreira 2006). The number of research papers published in recent years on its pharmacological properties, clinical therapeutic effects, and toxicity is evidence of the importance of AmB in contemporary medicine (Brajtburg et al. 1990; Tiphine et al. 1999; Fanos and Cataldi 2000; Lemke et al. 2005; Fanos et al. 2007; Moen et al. 2009; Hamill 2013). AmB, a metabolite of *Streptomyces nodosus*, causes disintegration of the fungal lipid membranes. These membranes contain ergosterol, which, similar to cholesterol, changes dynamic properties and stabilizes lipid bilayer structure. AmB has better selectivity for membranes containing ergosterol than for those containing cholesterol. This property enables use of the drug to treat deep fungal infections that occur in the aftermath of AIDS or transplantation. Despite its antifungal activity, AmB has many sides effects which are most probably related to AmB–cholesterol interactions (Wilcock et al. 2013). In addition, AmB has several side effects because of formation of aqueous pores (Cohen 1998); among these, nephrotoxicity (Fanos and Cataldi 2001) and hematotoxicity (Wong-Beringer et al. 1998) are the most serious.

**Fig. 1:**
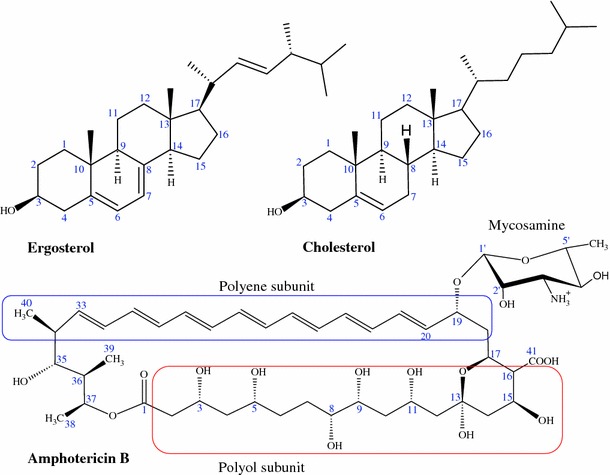
Schematic representation of sterols and amphotericin B

The AmB molecule comprises a macrolactone ring, which is β-glycosylated at position C19 with a mycosamine group (Ganis et al. 1971; Jarzembska et al. 2012). The ring is an almost flat chromophore with seven conjugated double bonds in the trans conformation. The ring also contains a more flexible polyol subunit (Fig. 1). At positions C13 and C17, the macrolactone ring contains a hemiketal ring. The presence of a carboxyl group at C16 and an amino group in the mycosamine head group determines the amphoteric character of this molecule. In addition, the specific AmB three-dimensional structure which has well defined hydrophobic and hydrophilic regions is responsible for its amphipathic properties. Consequently, AmB is poorly soluble in highly polar and apolar solvents. For this reason, AmB tends to aggregate (Shervani et al. 1996) in highly polar solvents, for example water, which gives rise to a variety of models explaining its antifungal activity.

**Fig. 2:**
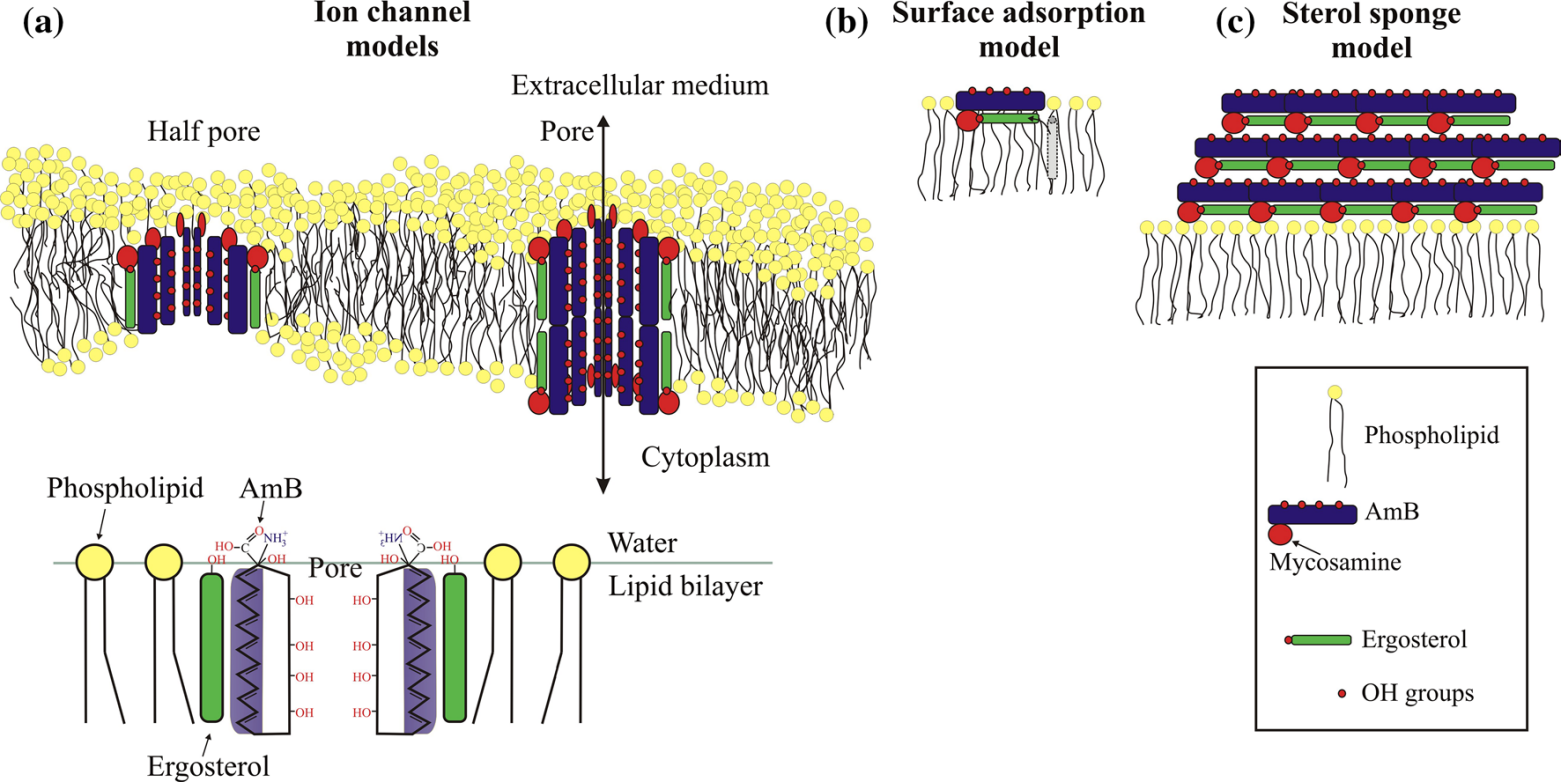
Models of amphotericin B function in phospholipid bilayers. **a** Classical-ion channel model in which AmB molecules aggregate in such a way that they form a barrel with their polyhydroxy chain groups pointing inward and their heptaene parts pointing outward. **b** Surface adsorption model in which AmB extracts ergosterol from the bilayer to the surface. **c** Sponge model in which large AmB aggregates extract ergosterol from the phospholipid membrane

Several possible mechanisms of action of AmB can be found in the literature. The first, oldest, and most studied is the ion-channel model proposed by Finkelstein and Holz (1973) (Fig. 2a). According to this model, AmB molecules aggregate in such a way that they form a barrel through a bilayer with their polyhydroxy chain groups pointing inward and the heptaene parts pointing outward. Pores can be created in both leaflets of the bilayer, or half-pores can be formed which bond two sides of the bilayer (Fig. 2a). The pore can be formed from different numbers of monomers, ranging from 4 to 12 (Cass et al. 1970; Gruszecki et al. 2003), and this has been confirmed by channel-conductivity experiments (Brutyan and McPhie 1996; Cotero et al. 1998). These pores are responsible for leaking of K^+^ ions and small organic particles vital for cell function. The second concept is based on the oxidative cell damage caused by amphotericin B (Brajtburg et al. 1985; Sokol-Anderson et al. 1988), which affects fungi and causes lysis of red cells. This effect induces formation of reactive oxygen species, for example superoxide, hydrogen peroxide, and hydroxyl radicals, which oxidize the lipid membrane (Lamy-Freund et al. 1985). AmB can bond to low-density lipoprotein receptors and probably modify their structure by oxidation (Barwicz et al. 1998, 2000). The oxidation damage induced by AmB can affect other cell functions not related to changes in cell permeability (Sokol-Anderson et al. 1986; 1988). AmB can also mediate killing of fungi cells by induction of a strong intracellular oxidative burst, as is observed for *Cryptococus neoformans*, which can be responsible for protein carbonylation (Sangalli-Leite et al. 2011). The third model is based on AmB surface adsorption in which antibiotic molecules oriented parallel to the plane lipid surface destabilize the membrane by sequestering ergosterol to the bilayer surface (Fig. 2b) (de Kruijff and Demel 1974; Mouri et al. 2008). The last concept is the sterol sponge model in which AmB exists as a large aggregate in the proximity of the fungal membranes which extract ergosterol from it (Anderson et al. 2014). In this process, strong interaction between ergosterol and AmB is a crucial (Palacios et al. 2011; Gray et al. 2012).

To better understand the interactions between AmB and living cells, it is important to understand how AmB interacts with lipids, including the arrangement of AmB in lipid–sterol environments (Matsumori et al. 2006; Gagoś and Arczewska 2010), the function of fungal cell walls, and the effects of AmB aggregation in biological systems (Barwicz et al. 1992; Barwicz and Tancrede 1997). Only a full understanding of these phenomena can lead to the design of forms of AmB with lower toxicity and greater efficacy (Bolard et al. 1980b; Bolard and Cheron 1982; Paquet et al. 2002; Matsuoka and Murata 2003; Sternal et al. 2004; Gabrielska et al. 2006; Hac-Wydro and Dynarowicz-Łątka 2006; Foglia et al. 2011), not only by appropriate formulation (Brogden et al. 1998; Andres et al. 2001; Hac-Wydro et al. 2005c; Menez et al. 2006; Moen et al. 2009; Hamill 2013; Pham et al. 2013) but also by molecular modification (Hac-Wydro et al. 2005c; Paquet and Carreira 2006; Czub et al. 2009; Croatt and Carreira 2011; Tevyashova et al. 2013; Wilcock et al. 2012, 2013). For instance, toxicity can be reduced by using appropriate cationic derivatives of AmB (Slisz et al. 2004). Other AmB modifications reveal that –OH groups in positions C8 and C9 or positions C7 and C10 give the most active forms of AmB, whereas forms with –OH groups at the C7 and C9 positions (Fig. 1) had less antifungal activity (Tevyashova et al. 2013). Applying modifications to one of the many –OH groups in AmB can affect ion transport through ion channels (Wilcock et al. 2012). Fluorescein–AmB conjugates can be a powerful tool for observing biological processes in living cells (Zumbuehl et al. 2004a), and tryptophan–AmB conjugates can increase channel activity in the absence of sterols (Zumbuehl et al. 2009). Recently synthesized AmB derivatives, and their antifungal properties and toxicity are widely described elsewhere (Baginski et al. 2006; Slisz et al. 2007; Baginski and Czub 2009).

Because of the substantial number of publications on medical formulations and clinical applications of AmB, this review concentrates on the most important discoveries related to the above mentioned questions reported in the literature of the last decade.

### AmB sterol binding

**Fig. 3:**
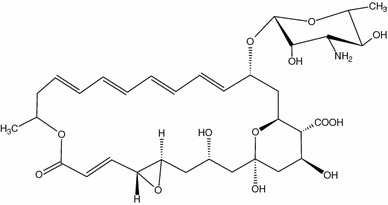
Structure of natamicin, another antifungal polyene antibiotic which is unable to create pores through cell membranes (is too short) but binds ergosterol in a similar manner to AmB

The generally accepted mechanism of action of AmB is based on the effects of both ergosterol binding and pore formation. According to Yilma et al. (2007), Palacios et al. (2011), and Gray et al. (2012), sterol binding is necessary for antifungal activity and AmB channel formation is only one of several sterol-binding-dependent mechanisms of action. For example, the antifungal compound natamycin (Fig. 3), which is shorter in length than AmB, was recently reported to bind ergosterol in yeast cell membranes without pore formation (te Welscher et al. 2008). This effect, inter alia, led the Burke group to the conclusion that the main factors responsible for AmB antifungal activity must be related not to pore formation but rather to binding of ergosterol (Gray et al. 2012). This concept was extended in the next work of this group (Anderson et al. 2014). According to authors, extramembranous AmB aggregates work as sponges extracting ergosterol from the fungal membranes (Fig. 2c). This is, overall, a coherent mechanism which agrees with biological observations of large AmB aggregates or AmB-rich structures (Strachecka et al. 2012). However, in both sets of experiments the ergosterol-to-lipid ratios, i.e. 1:10 (Gray et al. 2012) and 1:40 (Anderson et al. 2014), are significantly lower than those observed in natural systems; in *S. cerevisiae*, for example, this ratio is 30:70 (Schneiter et al. 1999). Alteration of the sterol-to-phospholipid ratio in fungal cell membranes is an established mechanism in the development of AmB resistance (O’Shaughnessy et al. 2009). As a result, it is vital to use a sterol-to-phospholipid ratio that models that found in AmB-sensitive fungal membranes rather than a ratio that suits the experimental technique used. In mammals, moreover, cholesterol is available in large amounts; such sponges can therefore be saturated with cholesterol much earlier than those in contact with fungal cell walls. It can easily be calculated thermodynamically that the AmB–cholesterol/AmB–ergosterol balance in situations in which cholesterol is in excess will be shifted to the advantage of AmB–cholesterol; thus, ergosterol extraction will be greatly limited. Moreover, living fungi have cell walls constructed of chitin, which is hydrophilic. This is a serious kinetic barrier to transport of ergosterol through fungal cell walls to the AmB super aggregates. The pore model is free from such effects and thus seems an attractive proposition.

**Fig. 4:**
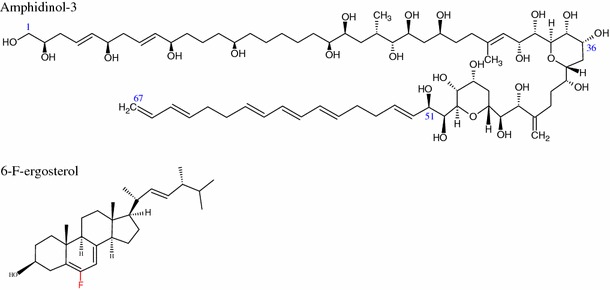
Structures of amphidinol-3 and 6-F-ergosterol. This modification inhibits interactions with AmB but not with another polyene antibiotic, amphodinol-3

Molecular dynamics simulations confirm that in the membrane environment AmB interacts with ergosterol 3–4 times more strongly than in solution, and this could be responsible for the more effective AmB assembly leading to functional transmembrane channels (Neumann et al. 2009, 2010, 2013a). In the first of these references the system consists of AmB–sterol complexes embedded in a sterol–DMPC system with a sterol content of 25 %. In the second reference the system is a DMPC bilayer with a sterol content of 30 % and in the last reference DPMC containing 0 and 30 % sterol is studied. The last study shows that AmB–cholesterol bonding is weaker not only because of weaker van der Waals (vdW) interactions compared with ergosterol, but also because of entropy reduction associated with a decrease in the conformational flexibility of the sterol side-chain. The significant effect of vdW interactions was confirmed by introducing a fluorine atom at the C6 position in ergosterol (Kasai et al. 2011). The fluorine weakens the sterol interaction with AmB, which is not observed for another antifungal antibiotic, amphodinol-3 (Fig. 4). The weaker interaction between AmB and cholesterol compared with that between AmB and ergosterol leads to the different behavior of this antibiotic in monolayers containing these sterols (Saint-Pierre-Chazalet et al. 1988; Seoane et al. 1998, 1999a, b; Miñones et al. 2005; Chang et al. 2010). These differences are also observed for modified AmB molecules (Hac-Wydro et al. 2005a, b, c; Baginski et al. 2006).

### Effect of the mycosamine group

It is known that the mycosamine group in AmB is important in the sterol-binding process, as is confirmed by isothermal titration calorimetry (Wilcock et al. 2013). If the group is removed, AmB binds neither ergosterol nor cholesterol and loses its antifungal properties. On the basis of the studies mentioned above and the fact that mycosamine has only been found in polyene macrolide natural products, this polyene–glycoside linkage serves as a sterol-binding group. Interestingly, the glycoside subunit is very similar to the main component of the cell walls of fungal chitin (Ramanandraibe et al. 1998), which is not observed in mammals. The C2′-OH from the mycosamine subunit is of major importance in the binding of AmB to the 3β hydroxyl group of cholesterol and ergosterol (Matsumori et al. 2005) (Fig. 1). However, when the OH group bonded to C2′ from mycosamine (Fig. 1) is removed, cholesterol binding is substantially weaker whereas the ergosterol-AmB bond is still strong (Wilcock et al. 2013). These different bonding strengths are explained by authors on the basis of the different conformer types for deOAmB–ergosterol and deOAmB–cholesterol which results in weaker vdW interactions for deOAmB–cholesterol. The stronger AmB–ergosterol interaction compared with that with cholesterol is attributed to the double bond and the additional methyl group in this sterol (Vertutcroquin et al. 1983; Charbonneau et al. 2001; Baginski et al. 2002). This specific atomic pattern is responsible for the better matching of ergosterol to the AmB heptaene chain (Baran et al. 2009).

### Effect of ergosterol concentration

A recent study has shown that the antifungal mechanism is based mostly on AmB binding to ergosterol which affects vital cellular functions in yeast-like endocytosis, vacuole fusion, pheromone signaling, and control of the activity of membrane proteins, among others (Gray et al. 2012; Palacios et al. 2011). However, no correlation has been observed between the level of ergosterol in different clinical yeast isolates and the antifungal activity of AmB (Gomez-Lopez et al. 2011) which is in contrast with results obtained for artificial membranes, for which maximum AmB activity was observed for 10 mol% ergosterol (Teerlink et al. 1980) and for which an increase in the amount of ergosterol in the monolayer promoted AmB incorporation (Barwicz and Tancrede 1997). The lack of correlation between the level of ergosterol in cell membranes and AmB antifungal activity can be related to different mechanisms of resistance of living yeasts (Sanglard and Odds 2002), different sterol compositions (Seitz et al. 1979; Brun et al. 2004; Vandeputte et al. 2008, 2011), greater membrane fluidity (Younsi et al. 2000; Venegas et al. 2003), and, most probably, the effect of cell walls (Ramanandraibe et al. 1998). This shows that artificial membranes are only an approximation of natural ones and results from such systems should be compared with those from natural ones with care.

### AmB aggregation

Even if the pore formation by AmB through the bilayer is of secondary importance, it is still an important aspect of its antifungal activity. Because aggregation of AmB in the lipid matrix is a crucial step in the barrel–stave channel formation, it is crucial to fully understand this process. A substantial number of experiments have been conducted with use of a wide range of techniques.

Hargreves et al. investigated the effect of aggregation of AmB in phospholipid nanodiscs (ND). They found that at a concentration of 2.5 mg AmB per 10 mg phospholipid AmB occurs in self-associated forms, but below this concentration the AmB occurs as the monomeric form observed in solvents (Hargreaves et al. 2006). This is in accordance with a previous study in which aggregated forms of AmB were observed when the ratio of AmB to lipid molecules was higher than 1:1,000 (Fujii et al. 1997). Below this concentration, AmB exists in the lipid bilayer mostly in the monomeric form. Gruszecki et al. (2009) showed that AmB forms aggregated as dimers in pure lipid bilayers and lipid bilayers containing cholesterol, whereas in bilayers containing ergosterol both monomeric and aggregated forms are present. This dynamic molecular study also confirmed that AmB forms dimers in lipid bilayers with and without sterols, but in the presence of ergosterol AmB–AmB interaction is less favorable (Neumann et al. 2013a). This was also confirmed by fluorescence lifetime imaging microscopy of monomolecular layers formed at the argon–water interface deposited on to a glass support by the Langmuir–Blodgett technique. In this situation only monomeric forms of AmB were observed for monolayers containing ergosterol (Gruszecki et al. 2009). Comparison between FTIR and Raman spectra obtained for crystalline and amorphous AmB reveals that in lipid environments AmB aggregates and/or dimers have a similar arrangement to that observed in mono crystals, which is characterized by a ~1,010 cm^−1^ band (Gagoś et al. 2012). For amorphous AmB obtained by DMSO evaporation, in which AmB molecules are randomly oriented, this band is not observed. The presence of the aggregated forms in DMPC bilayers containing ergosterol is also confirmed by solid-state NMR (Matsumori et al. 2006). The greater number of AmB monomers is related to the stronger interaction of AmB with ergosterol, which is responsible for AmB monomerization. Circular dichroism (CD) experiments reveal that AmB can already associate in water solutions in the range 5 × 10^−7^–10^−4^ M (Mazerski et al. 1982). The AmB aggregation process also occurs in monocomponent monolayers formed at the argon–water interface. Under these conditions spontaneously formed dimers with homogeneous distribution are observed in the monolayer. These dimers can assemble as higher oligomers which are most probably responsible for channel formation (Gagoś and Gruszecki 2008). Brewster angle microscopy has shown that at low surface pressure the surface area of AmB in the expanded state (0.4 mN/m) is ~180 Å^2^, which corresponds to horizontal AmB molecules. In the condensed state, the surface area is 55 Å^2^, which corresponds to vertically oriented AmB molecules. Transition from one orientation to the other is continuous at plateau surface pressure (Minones et al. 2001). AmB molecules on a water surface are most probably in the aggregated form. Diezi and Kwon (2012) measured the process of aggregation of AmB in 1,2-distearoyl-*sn*-glycero-3-phosphoethanolamine-*N*-(methoxy(poly(ethylene glycol))-5000 (ammonium salt) (PEG–DSPE) micelles. They found that AmB in the presence of ergosterol is aggregated whereas in the presence of cholesterol or pure PEG-DSPE it is mostly in the monomeric form. This seems contrary to results presented earlier. However, this system differs substantially from that described above and AmB aggregation can occur differently. In this case, the amphiphilic character of AmB could be of crucial importance in interactions with these specific environments (PEG polymer), which can, in turn, change the aggregation behavior of AmB.

In work by Gruszecki et al. (2012) fluorescence techniques were used to detect dimers and aggregates of AmB in different environments. However, according to studies by Bolard et al. (2009, 2011) those bands should not be related to dimers and/or aggregates of AmB. A recent study has shown that characteristic changes of electronic absorption spectra previously related to formation of AmB dimer and/or oligomer aggregates exactly overlap bands related to the oxidized forms of AmB (Gagoś and Czernel 2014). The appearance of oxidized AmB forms in systems measured in air is not surprising, because Ganis et al. (1971) had already noted that AmB is sensitive to oxidation. Therefore, atmospheric oxygen may be sufficient to oxidize double bonds in AmB molecules in the presence of light. This simply suggests that several spectroscopic experiments in which the AmB was not appropriately protected against oxidation should be repeated or at least reinterpreted.

### AmB pore formation

**Fig. 5:**
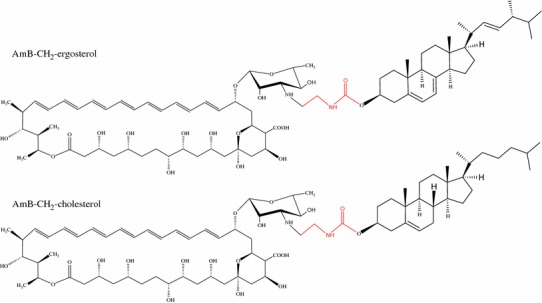
Structures of AmB–sterol linking. In this case, only the link built of two carbon atoms is shown; in the original study a link containing 6 carbon atoms was also studied

Yang et al. (2013) measured the effect of AmB on pore formation in membranes containing ergosterol by use of fluorescent dyes of known average diameter. They showed that increasing AmB concentration tends to increase the preferential accumulation of AmB ion channels in membranes. It has also been found that membrane pores can be formed not only in the presence of sterols but also without them (Cotero et al. 1998). It is important to mention that channel formation depends on AmB concentration, and the presence of sterols is not necessary for this process (Fujii et al. 1997). Addition of sterols also affects the dwell time of artificial AmB channels in the patch-clamp technique, which is longer for cholesterol-containing membranes (ions occupy channels for a longer time, thus blocking them) than for ergosterol-containing membranes (Matsuoka and Murata 2002). For that reason, ion transport through channels created in the presence of ergosterol is more efficient than that in the presence of cholesterol (Ostroumova et al. 2012). The authors suggested that the process of association and/or dissociation of channel-forming molecules depends on membrane fluidity. This is in agreement with previous studies in which sterols did not directly affect pore formation but rather affect the membrane structure which produces a different threshold for the formation of AmB channels (Cotero et al. 1998). Results similar to those of Matsuoka and Murata (2002) have been obtained for sterols linked covalently to AmB (Fig. 5). In this case, the probability of the channel being open was greater for ergosterol-linked AmB than for cholesterol-linked AmB (Matsumori et al. 2004). This can be explained on the basis of results from a study of neutron diffraction of multibilayers (Foglia et al. 2012) (sterol–lipid ratio 30:70), which revealed formation of full AmB pores in the lipid bilayer. Detailed modeling shows that cholesterol and AmB in AmB–POPC–cholesterol bilayers are wholly contained within the separate leaflets of the bilayers. For AmB–POPC–ergosterol, both AmB and ergosterol intrude from one leaflet to the opposite one. Such connectivity between half-pores can stabilize the transmembrane ion-channel structure and thus increase its permeability. Interestingly, insertion of AmB in the POPC–cholesterol bilayer causes a 3-Å shift in the position of cholesterol, whereas for POPC–ergosterol the shift is only 0.5 Å. Because this is only the scattered length density profile of all the atoms in the system, this might indicate reorientation of the mycosamine group in the bilayer, as has been suggested by the authors and additionally by work of Matsumori et al. (2005). Similar to the neutron data, surface X-ray scattering data also indicate vertical insertion of AmB into the lipid monolayer (Kamiński et al. 2014). It should be noted that the article by Gagoś et al. (cited in Foglia et al. 2012) relates to the DPPC not POPC lipid system. In general, the effect of AmB and nystatin on ion permeability is much stronger when lipid membranes contain ergosterol. Permeable ion-channels are also formed in the presence of cholesterol, as was investigated by Yilma et al. (2007) for a cholesterol monolayer. Similar to Sykora et al. (2004) and, they found that AmB and cholesterol form a complex with of stoichiometry 2:1, but Yilma et al. also showed that AmB in the presence of cholesterol assembles in three, four, etc., subunit aggregates which form ion channels. According to the above-mentioned studies, it is most probable that the AmB–cholesterol interaction is the main factor responsible for toxicity to mammalian cells whereas the interaction between AmB derivatives and lipids is less important for toxicity.

Formation of cation-selective ion channels by AmB in a model lipid membrane and in membranes of eukaryotic cells is reviewed in detail by Cohen (2010). This review also concentrates on the effect of membrane thickness, the types of sterol used, and the sterol-rich lipid rafts on the pore-formation process, and led the author to develop a cell model that serves as a framework for understanding the intracellular K^+^ and Na^+^ concentration changes induced by the cation-selective AmB channels. Ion-channel selectivity has also been observed for new conjugates bearing a calixarene structure covalently linked to four AmB molecules (Paquet et al. 2006). These macro molecules adopt a cone conformation that mimics the structure of membrane pores. These artificial pores have similar properties to those created naturally by AmB in membranes, which strongly supports the generally accepted classical AmB pore model.

### AmB channel diameter

Interestingly, the channels created by AmB in erythrocyte membranes (with cholesterol) are in the range 0.36–0.37 nm as measured in a conduction osmotic protected experiment (Katsu et al. 2008). The channel diameters measured by Katsu et al. (2007) for liposomes composed of egg phosphatidylcholine and cholesterol in a conduction osmotic protected experiment are also in a similar range, 0.36–0.46 nm. For ergosterol-rich membranes the diameter is in the range 0.4–1.0 nm (Reeves et al. 2004). This indicates that the sterols do not significantly affect channel diameter and thus the subtle effect of better conductance must be related to sterol distribution in the proximity of the AmB molecules (Neumann et al. 2013b), which affect the electric field inside the channel and consequently the dwell time, as already mentioned. The measured diameters are similar to that measured earlier by atomic force microscopy (AFM) (~0.6 nm) (Gruszecki et al. 2002, 2003). In this case, AFM should show the upper limit of pore diameter, whereas osmotic techniques underestimate pore size because of intermolecular interactions. By use of different fluorescence dyes, Yang et al. (2013) demonstrated that in ergosterol-rich membranes (bilayers) the pore size (diameter) depends on AmB concentration. At low AmB concentrations (50 pg/ml) this is approximately 0.16 nm whereas for AmB concentrations of 2 ng/ml the pore diameter is nearly 16 nm. From this experiment it is clear that the concentration of AmB in bilayers is of primary importance in determining pore size, whereas sterols have a minor effect only. The distance between AmB dimers in the barrel–stave ion channel corresponds to the distance between covalent dimers of AmB, which is 6.9 Å, measured for multilamellar vesicles by use of solid-state NMR (Umegawa et al. 2012a). This distance is significantly shorter than that previously measured by the same group, i.e. 11–12Å (Kasai et al. 2008). According to the authors, this difference results either from covalent linkage, as a result of which AME (two linked AmB molecules) units are closer in the membrane assembly, or from inappropriate assumptions when estimating the intermolecular 13C–19F distance.

### Geometry of AmB–sterol associates

**Fig. 6:**
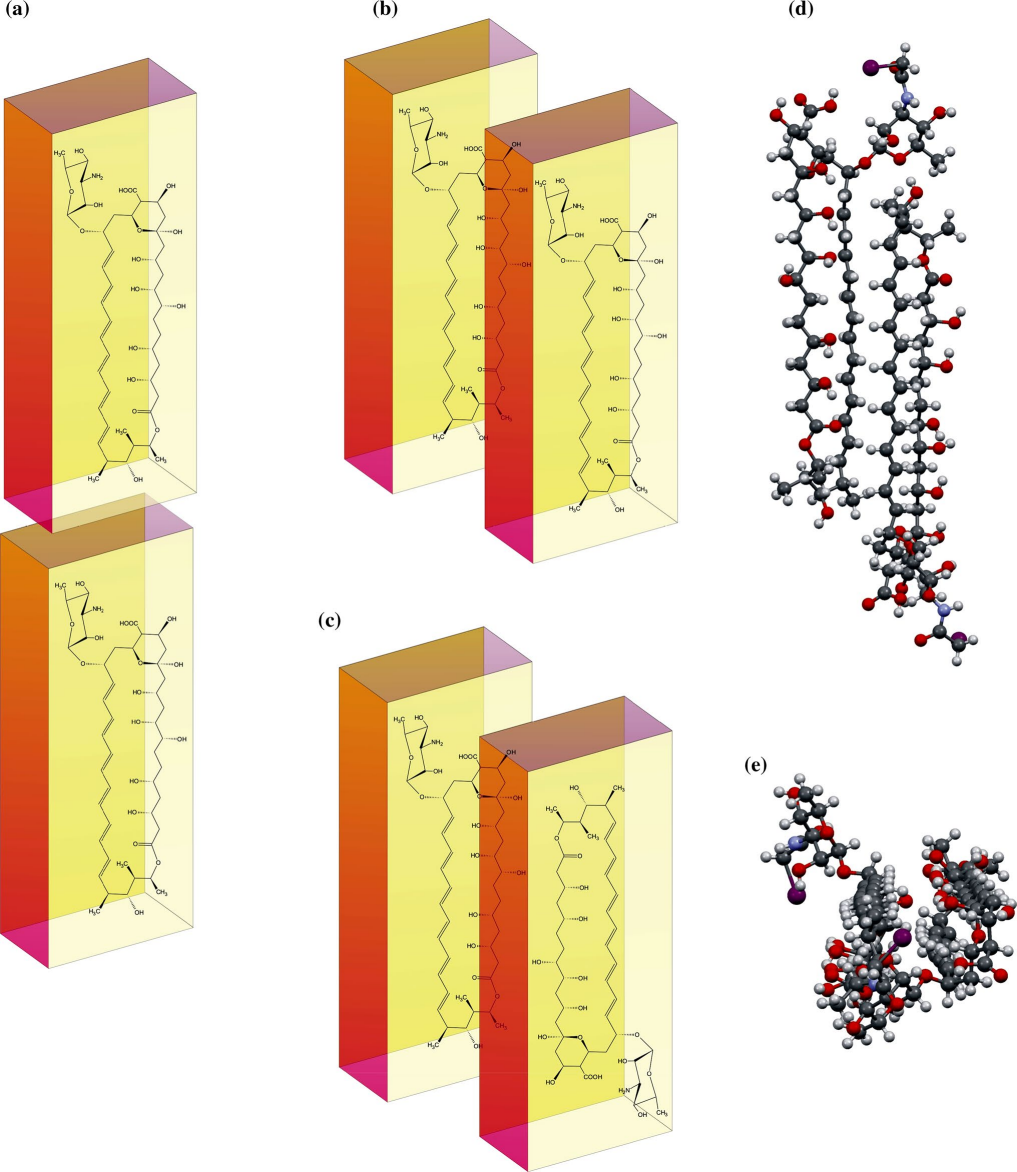
**a** Card-pack, **b** head-to-head, and **c** head-to-tail orientation of AmB molecules in a dimer. **d** Iodoacetyl AmB (AmB-I) molecules packed into a crystal lattice [11]. **e** Side view of the same structure. It can be seen that AmB-I molecules have head-to-tail orientation and the polyol subunit is in contact with the heptaene chain

Molecular dynamics simulations in water environments suggest that AmB forms AmB–ergosterol–AmB (2:1 stoichiometry) associates with head-to-head interaction between AmB and ergosterol (Baran et al. 2009). The calculated stability of this complex was substantially higher than that of 1:1 stoichiometry. The energy of the complex is highly dependent on the surrounding environment, and for water the head-to-head conformation is preferable. As might be expected, AmB–ergosterol complexes can behave differently in a lipid bilayer part of which is hydrophobic. A solid-state NMR study of multilamellar vesicles conducted by Umegawa et al. (2008, 2012b) suggests that both head-to-tail and head-to-head conformations are possible (Fig. 6; POPC–sterol vesicles containing 10 % sterol). The head-to-tail conformation of AmB–ergosterol requires that the AmB dimer is also in the head-to-tail conformation and thus the orientation of ergosterol relative to both AmB molecules should be the opposite. In vivo, AmB is delivered from outside the cell only, and the most reasonable way of entering the membrane is by embedding the less polar part of AmB (i.e. the tail) into the lipid environment from water. The head-to-tail conformation is much less preferable. Another explanation is to form an AmB–ergosterol–AmB complex in the head-to-tail conformation compared with AmB–AmB molecules by diffusion of the binary complex of AmB–ergosterol up and down in the membrane. If another single AmB molecule can move from one lipid leaflet to another (without flip-flop), then sterols from this second leaflet can interact with AmB in a head-to-tail manner. A similar result can be achieved when a binary AmB–ergosterol complex moves from one leaflet to another without flip-flop and meets a single AmB molecule in the right conformation. However, in this case also, floating from one lipid leaflet to another is very likely. The idea that AmB–AmB head-to-tail structures can be formed in membranes—especially in vivo—should therefore be accepted with caution.

Interestingly, quantum chemical calculations predict that the head-to-tail AmB dimer conformation has the lowest energy, but this is solely for isolated systems without any effect of solvent (Jarzembska et al. 2012). A similar conformation was also observed by Barwicz et al. (1993) in AmB aqueous solution. They recorded bathochromic and hypsochromic shifts in spectra which are associated with “card pack” and “head-to-tail” AmB patterns. The latter was interpreted by authors as an effect of dimerization along the longest molecular axis of AmB, whereas the former is the effect of interactions between molecules responsible for hydrophobic pore formation. These results are apparently in contrast with the molecular dynamics study from the bilayer where head-to-head should be dominant (Neumann et al. 2013b) (DMPC bilayer with 30 % sterols). These differences can be simply explained as the effect of different environments. AmB, as an amphiphilic molecule, can form, or even should form, totally reverse associates or structures in non-polar (lipids acyl chains) versus polar (water) environments. The lack of any environment in quantum chemical calculations can substantially affect the total energy of dimers of this size and thus lead to mistaken conclusions. Other experimental studies suggest that both forms of AmB—head-to-tail and head-to-head (tail-to-tail)—occur naturally in a lipid environment when the concentration is high enough to enable this process (Volmer and Carreira 2010; Hirano et al. 2011).

### Interaction of AmB with lipids

It is obvious that phospholipids are always associated with sterols in biological membranes; therefore, when analyzing sterol–AmB interactions, the effect of the lipids must also be taken into account (Bolard 1986a, b; Rapp et al. 1997; Dynarowicz-Łątka et al. 2003; Hac-Wydro and Dynarowicz-Łątka 2006). Usually, the interaction leads to the formation of AmB complexes, as for dipalmitoyl phosphatidylserine (DPPS), for which the stoichiometry between AmB and DPPS is 2:1 (Minones et al. 2003). The optimum stoichiometry for an the AmB–DPPC system is also 2:1, with two horizontally oriented AmB molecules and one DPPC molecule in a vertical position, as proposed by Minones et al. (2002). The interaction between AmB and phospholipids on formation of ion channels across a biomembrane was investigated by Matsuoka et al. (2003), by using their covalent conjugates. As might be expected, the membrane-permeabilizing activity was substantially affected by the chain lengths of the fatty acyl groups of the phospholipids. Acyl chain length has a direct effect on bilayer thickness; it can also affect the lipid–AmB interaction and, as a consequence, ion channel assembly. The different interaction of AmB with lipid membranes in vesicles was also observed for the (*S*) and (*R*) enantiomers of POPC (Jeon and Carreira 2010). The kind of lipid used and its fluidity have a substantial effect on pore formation (Ostroumova et al. 2012). It has also been found that AmB interacts differently with egg yolk phosphatidylcholine, dimyristoyl (DMPC), and dipalmitoyl phosphatidylcholine (DPPC) phospholipid bilayers (Bolard et al. 1980a, b). The double bond of egg yolk phosphatidylcholine affects lipid fluidity, which has a direct effect on AmB pore formation in bilayers. It was also found that AmB can occur in different aggregation forms in lipid bilayers. Marty and Finkelstein (1975) suggested that AmB can form pores and half pores, that sterols play a minor role, and that pore formation is prevented by some lipids.

The different lengths of phosphatidylcholine saturated acyl chains (ranging from 14 to 22 carbon atoms) and the presence of cholesterol affect the conformation of AmB in bilayer vesicles (Bolard and Cheron 1982). For POPC vesicles containing sterols, bilayer thickness was affected not only by the kind of lipid used but also by the concentration of AmB. Ions can also affect the AmB–lipid interaction. Arczewska and Gagoś (2011) found that AmB has a greater affinity for DPPC in the presence of K^+^ than in the presence of Na^+^. The most stable monolayers in the presence of both ions were formed by AmB and DPPC with 1:1 and 2:1 stoichiometry. The FTIR spectra revealed that the ionic state of AmB (which is a function of pH) and the presence of sterols led to changes in membrane fluidity and the molecular packing of the AmB molecules in the lipid membranes (Gagoś and Arczewska 2012). In this way, pH can affect not only AmB but also liposomes. According to these authors, both pH and the presence of sterols affect pore formation. Thus, it is important to conduct such experiments at physiological pH.

### Orientation of AmB in membranes

Electron spin resonance spectroscopy revealed that AmB orientation in lipid model membranes is a two-step process (Man and Olchawa 2013). At a concentration of 0.25–0.5 % AmB molecules initially lie flat; at higher concentrations, ca. 2.5–3 %, in a monolayer re-orientation to the vertical position occurs. This vertical orientation of AmB is responsible for channel formation and bilayer perforation. In this orientation AmB interacts strongly with the lipid head groups and restricts the molecular motion of choline (Gabrielska et al. 2006). Fourier-transform infrared spectroscopic (FTIR) study of deposited lipid (free and with sterols) monolayers after binding AmB from the water subphase revealed that most of the AmB molecules bound to the membrane were located in the polar head groups or interacted with them. In pure DPPC and DPPC containing cholesterol, the distribution between vertically and horizontally oriented AmBs is similar. However, in the presence of ergosterol the dominant form of AmB is horizontal (Gagoś et al. 2005). The possibility cannot be excluded that the transition of the monolayer on the solid substrate had an effect on the position and orientation of AmB, but a strong interaction between AmB and ergosterol was still observed. It is also possible that the more rigid layers containing ergosterol were less accessible to AmB, which, therefore, accumulated under the monolayer parallel to the surface and after transfer to the solid substrate was between the substrate and polar head groups of the lipid. Even more surprising results were obtained from a molecular dynamics study in which AmB preferentially took a vertical position, perpendicular to the membrane surface of dimyristoyl phosphatidylcholine (DMPC), with no propensity to enter the membrane (Sternal et al. 2004). The system on which the calculation was performed consisted of 200 molecules of DMPC, one molecule of AmB and 8,065 water molecules. At initialization, AmB was placed on the bilayer surface. According to the modeling, it is very likely that a single molecule enters the membrane, which suggests that AmB has to be at least in the dimeric form to enter the bilayer. The latest X-ray gas–water interface diffraction studies performed for DPPC monolayers show that AmB is incorporated into a monolayer, perpendicular to the surface, into both hydrophobic and hydrophilic parts of the lipid. This also occurs in the presence of cholesterol and ergosterol but, in contrast with the surface pressure study (Dynarowicz-Łątka et al. 2005) the amount of AmB incorporated when these two sterols are present is the same and depends on the surface pressure (Kamiński et al. 2014). For cholesterol, this apparent discrepancy is simply explained by monolayer corrugation and/or buckling or roughening which reduces the surface pressure. For the DPPC–ergosterol system, the monolayer is much less corrugated. The same orientation of AmB was observed in multibilayers investigated by neutron diffraction (Foglia et al. 2012).

### Effect of lipid rafts

Biological membranes are not flat and homogenous as was imagined in the early 1970s (Singer and Nicolson 1972). The contemporary model is heterogeneous not only in structure but also in composition. The presence of sterol-rich micro domains in the liquid ordered phase which freely float in a less sterol-rich liquid disordered phase, affect cellular transport and signal processes (Quest et al. 2004; Simons and Ikonen 1997). Such sterol-rich domains are called rafts. Ordering of lipid chain conformations is induced in rafts with a large sterol content (Fournier et al. 2008). This lipid ordering affects the affinity of AmB for liposomes, which in the solid phase is higher than in the liquid phase (indirect effect of sterol) (Bolard et al. 1981; Coutinho and Prieto 1995; Zumbuehl et al. 2004b). In these circumstances sterols contribute to ordering of aliphatic lipid chains, and ordering for ergosterol is greater than for cholesterol, because of its greater rigidity (Urbina et al. 1995; Hsueh et al. 2005; Czub and Baginski 2006). This is also true in the presence of AmB (Fournier et al. 2008). In the gel phase, AmB does not change the conformational order of lipid hydrocarbon chains; however, in the fluid phase the drug affects the structure of the lipid environment. According to the authors, AmB can initiate in-plane ergosterol redistribution, which is not observed for cholesterol. This is in accordance with an H^1^ NMR study which shows that AmB, in the presence of ergosterol, interacts more strongly with the aliphatic lipid chains, whereas this is not observed for lipids containing cholesterol (Gabrielska et al. 2006). This is supported by the study by Umegawa et al. (2008), in which a substantial increase in the distance between AmB molecules was observed. For bilayers with cholesterol, no significant AmB–AmB distance changes were recorded, which must be related to its different location in the bilayer in comparison with the ergosterol system. Solid-state NMR experiments not only show that ergosterol interacts with AmB more strongly than with cholesterol in lipid environments but also the presence of ergosterol significantly affects AmB mobility in lipid bilayers (Matsumori et al. 2009). In summary, AmB may be accumulated more efficiently in lipid rafts which are more ordered than the disordered liquid phase. This different sterol distribution in the lipid bilayer in the presence of AmB can obviously affect channel formation.

## Conclusion

Despite very extensive investigations over the last 40 years, the mechanism of action of AmB is still not completely understood. Several studies suggest the antifungal activity of AmB is related to the presence of ergosterol, the main sterol of fungal cells. Toxicity toward mammal cells rich in cholesterol is smaller, but still high enough to cause many side effects. The action of AmB seems more complex than was imagined in the early 70s. The new models of oxidation burst or sterol sponge seem to be equally important for the antifungal properties as the well-known pore model. However, the old channel model is still attractive, especially because it explains in a simple way the whole range of effects, for example ion conductivity, AmB diffusion into membranes, and observed interactions with both lipids and sterols in artificial membranes. It is also clear that membranes of living organisms are more complex than model membranes, and the toxic effect of AmB toward fungal cells can also be related to the presence of rafts and membrane proteins. Experiments such as those conducted by the Burke and Murata groups in which specially modified sterols, AmB, and lipids were used can shed more light on the mechanism of action of AmB. However, Raman or fluorescence confocal microscopy studies of living yeasts treated with unmodified AmB should verify the relevance of the different models, especially that of oxidation burst and ergosterol sponge.
